# The prevalence of severe depression in Iranian older adult: a meta-analysis and meta-regression

**DOI:** 10.1186/s12877-020-1444-0

**Published:** 2020-02-03

**Authors:** Nader Salari, Masoud Mohammadi, Aliakbar Vaisi-Raygani, Alireza Abdi, Shamarina Shohaimi, Behnam Khaledipaveh, Alireza Daneshkhah, Rostam Jalali

**Affiliations:** 1 Department of Nursing, School of Nursing and Midwifery, Kermanshah University of Medical Sciences, Kermanshah, Iran; 2 Department of Biology, Faculty of Science, University Putra Malaysia, Serdang, Selangor Malaysia; 3 Department of Psychiatric Nursing, School of Nursing and Midwifery, Kermanshah University of Medical Sciences, Kermanshah, Iran; 4 School of Computing, Electronics and Maths, Coventry University, London, UK

**Keywords:** Prevalence, Severe depression, Iranian older adult, Meta-analysis

## Abstract

**Background:**

Depression is one of the most common psychiatric disorders in the older adult and one of the most common risk factors for suicide in the older adult. Studies show different and inconsistent prevalence rates in Iran. This study aims to determine the prevalence of severe depression in Iranian older adult through a meta-analysis approach.

**Methods:**

The present meta-analysis was conducted between January 2000–August 2019. Articles related to the subject matter were obtained by searching Scopus, Sciencedirect, SID, magiran, Barakat Knowledge Network System, Medline (PubMed), and Google Scholar databases. The heterogeneity of the studies was evaluated using I^2^ index and the data were analyzed in Comprehensive Meta-Analysis software.

**Results:**

In a study of 3948 individuals aged 50–90 years, the overall prevalence of severe depression in Iranian older adult was 8.2% (95% CI, 4.14–6.3%) based on meta-analysis. Also, in order to investigate the effects of potential factors (sample size and year of study) on the heterogeneity of severe depression in Iranian older adult, meta-regression was used. It was reported that the prevalence of severe depression in Iranian older adult decreased with increasing sample size and increasing years of the study, which is significantly different (*P* < 0.05).

**Conclusion:**

Considering the high prevalence of severe depression in Iranian older adult, it is necessary for health policy makers to take effective control measures and periodic care for the older adult.

## Background

Older adult is an inevitable biological process that affects all living things and is associated with unpleasant experiences in some cases [1]. The aging process refers to the gradual decline in the function of the body’s systems, including cardiovascular, respiratory, genitourinary, endocrine glands, and immune system [2].

Social, economic, and scientific developments in recent years have increased life expectancy and reduced mortality rates, leading to an increase in the world’s older adult population [3]. The older adult population is estimated to be doubled during next 40 years in the world [4].

Today, aging has become a major global phenomenon and according to WHO statistics, the number of older adult in Southwest Asian countries, including Iran, will reach 15% of the total population by 2030 [5]. Other reports suggest that the number of people aged ≥60 years will increase from 841 million people in 2013 to 1.2 billion people in 2025, with 70% of them living in developing countries and 8 out of every 10 older adult people in the world are expected to live in developing areas by 2050 [6].

Iran, as one of the developing countries, is not excluded from these demographic changes, with the older adult population is projected to grow from 8.2% in 2011 to 10% in 2021 [7]. Despite the growth of the older adult population, the needs and problems of this stratum are more pronounced and need to be taken into consideration [7–9].

Depression is one of the most common psychiatric disorders in the older adult and one of the most common risk factors for suicide in the older adult [10], which accounts for nearly 24% of successful suicides, and older adult suicide victims attempted suicide in their first depression attack. The frequency of the symptoms of major depression has been reported to be 8–15% and approximately 30% among the non-hospitalized and hospitalized older adult, respectively [10–12].

Previous studies in different parts of Iran show different reports of the prevalence of severe depression, including 23% in Isfahan [13], 3.3% in Birjand [14], and 11% prevalence in the study in Gilan [15]; therefore, as can be seen, the reported information provides scattered and inconsistent information and since interventional studies to reduce the prevalence of severe depression in the older adult require accurate and consistent information in order to prevent severe depression problems and complications in this population, the question is, how much is the overall prevalence of severe depression in Iranian older adult?

The aim of the present study is to review the overall prevalence of severe depression in Iranian older adult based on a meta-analysis approach.

## Methods

This study is a meta-analysis, based on the findings of studies on the prevalence of severe depression among Iranian older adult, including articles published in domestic and foreign journals and searches carried out in the databases of Sciencedirect, Scopus, SID, magiran, Medline (PubMed), Barakat Knowledge Network System, and Google Scholar between January 2000 to August 2019. The search process was carried out using the keywords Older adult, Depression, Mood Disorders, Types of Depression, Severe Depression and their English equivalents and their possible combinations, search engine using both English and Persian words and (AND) and (OR) operators were used in combination to provide more comprehensive access to all articles, therefore, the OR operator was used to check for common names for disorders such as (Older adult OR Aging), (depression OR Depressive Disorder), (mood disorders OR Affective Disorders). The AND operator was also used among the keywords (Older adult AND depression AND severe depression) by matching words in the MeSH browser.

### Evaluation of articles and quality control

All articles were first collected using the selected keywords and a list of abstracts was prepared after the search was completed. After hiding the article specifications, including the name of the magazine and the author, the full text of the articles was made available to the reviewers. Each article was studied independently by two reviewers and if the article was excluded, the reason was mentioned. Articles in Persian and English extracted from cross-sectional studies on the prevalence and of severe depression in Iranian older adult met the inclusion criteria. Other review, case-control, cohort, and intervention studies were excluded from the list of articles. The studies were then reviewed based on four-phase PRISMA (2009.

STROBE checklist was used to review studies. This checklist consists of various methodological aspects, including study objectives, determination of appropriate sample size, type of study, sampling method, research population, data collection method, definition of variables and procedure, study data collection tool, study objectives, statistical tests, and findings. Accordingly, a maximum quality evaluation score of 32 was considered and articles with scores below 14 were excluded from the study.

### Statistical analysis

Data were analyzed using Comprehensive Meta-analysis software (Biostat, Englewood, NJ, USA version 3), the heterogeneity of the studies was assessed using the I^2^ test and probability of publication bias Funnel chart using Egger test and significance level 0.05 and also to investigate the effects of potential factors on heterogeneity of studies from Meta-regression test.

## Results

**Fig. 1:**
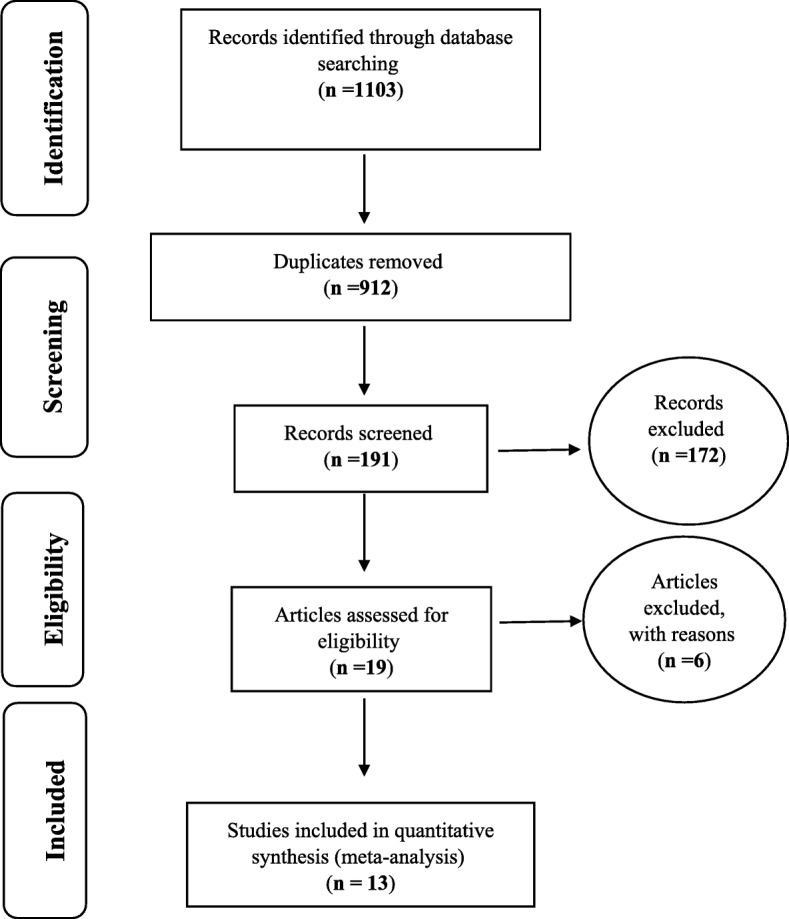
the flowchart on the stages of including the studies in the systematic review and meta-analysis (PRISMA 2009)

Based on investigations on the prevalence of severe depression in Iranian older adult, including articles published in domestic and foreign journals and searches in Magiran, SID, Barakat Knowledge Network System databases, Medline, ScienceDirect, Scopus, and Google scholar, PRISMA 2009 was used to show the reviewing process (Fig. 1). A total of 191 articles met the initial inclusion criteria based on initial reviews after deleting 912 duplicate articles. Ultimately 13 articles entered the meta–analysis phase after excluding 172 unrelated articles, 6 articles during secondary review because of lack of access to their abstracts and main articles, and low quality of articles (Table 1).

**Table 1:** Specifications of studies entered the study

Row	Author	Publication year	published	Area	Participants’ Age	Sample size	Prevalence
1	Sadeghi et al. [13]	2002	In Persian	Tehran	65–85	279	14.7
2	Rajabizadeh et al. [14]	2003	In Persian	Kerman	≥50	1212	0.3
3	Khodadadi et al. [15]	2007	In Persian	Guilan	64.6 ± 5.05	100	11
4	Sohrabi et al. [16]	2008	In Persian	Shahroud	76.5 ± 11.1	136	43.5
5	Manzori et al. [17]	2009	In Persian	Isfahan	60–74	248	23
6	Nejati et al. [18]	2009	In Persian	Qom	68.2 ± 7.7	151	46.3
7	Mobasheri et al. [19]	2009	In Persian	Shahr-e-kord	68.7 ± 16.1	61	3.5
8	Kashfi et al. [20]	2010	In Persian	Shiraz	≥60	120	9.1
9	Gharanjik et al. [21]	2011	In Persian	Torkaman	–	300	3
10	Ghaderi et al. [22]	2012	In Persian	Bukan	60–90	302	7
11	Miri et al. [23]	2015	In Persian	Birjand	63.6 ± 3.7	389	3.3
12	Khalili et al. [24]	2015	In Persian	Kashan	72.07 ± 9.03	500	5.8
13	Ashrafi et al. [25]	2017	In Persian	Salmas	69.9 ± 6.8	150	6.3

### Publication bias

**Fig. 2:**
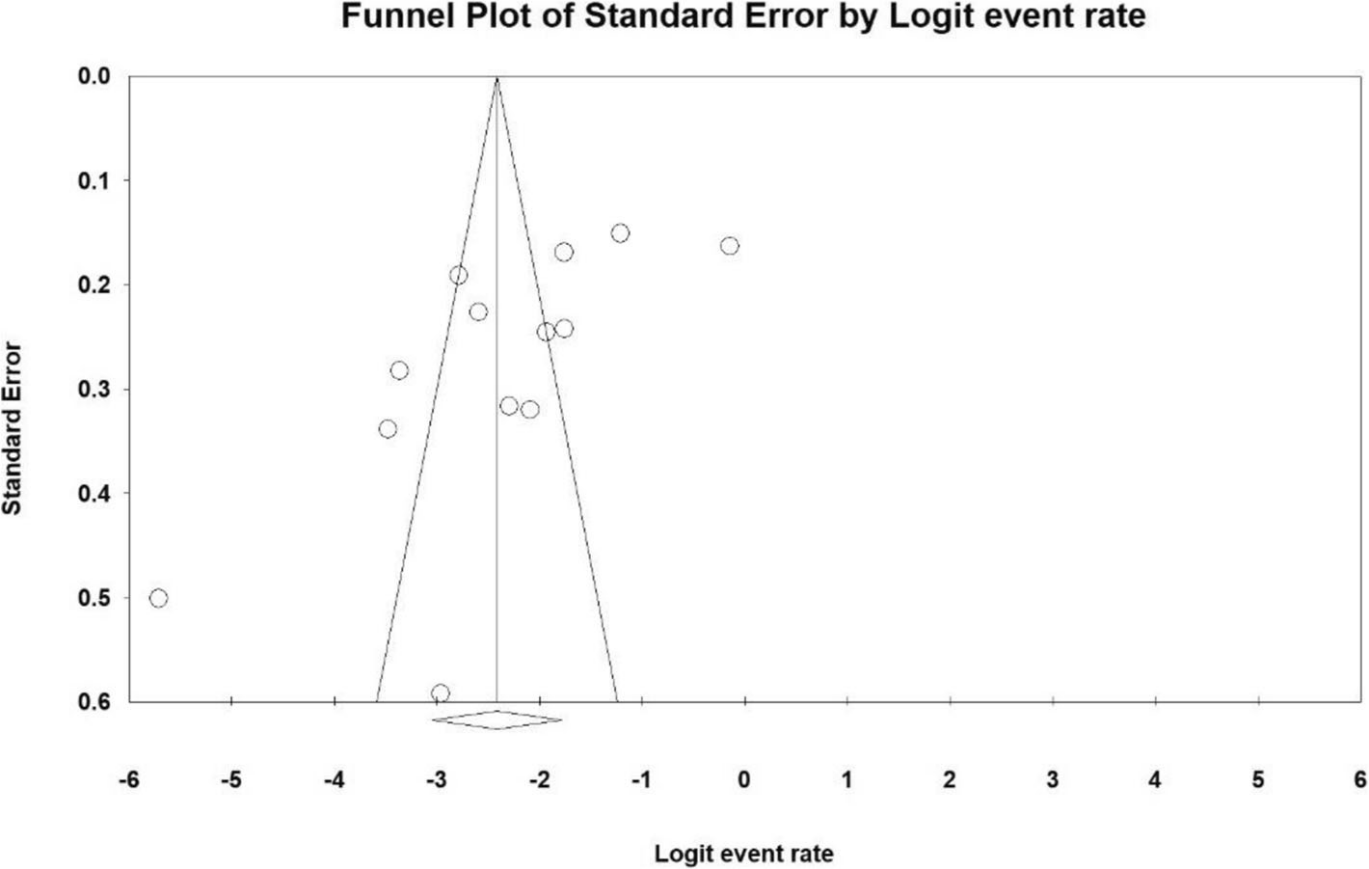
Funnel plot of results on the prevalence of severe depression among Iranian older adult

The heterogeneity of the studies was evaluated using I^2^ test that was equal to 98.7%, indicating high heterogeneity of the included studies, so, random effects model was used to combine the results of the studies. Also, the results of the publication bias were compared using Egger test (Fig. 2), which was not statistically significant (*P* = 0.061).

**Fig. 3:**
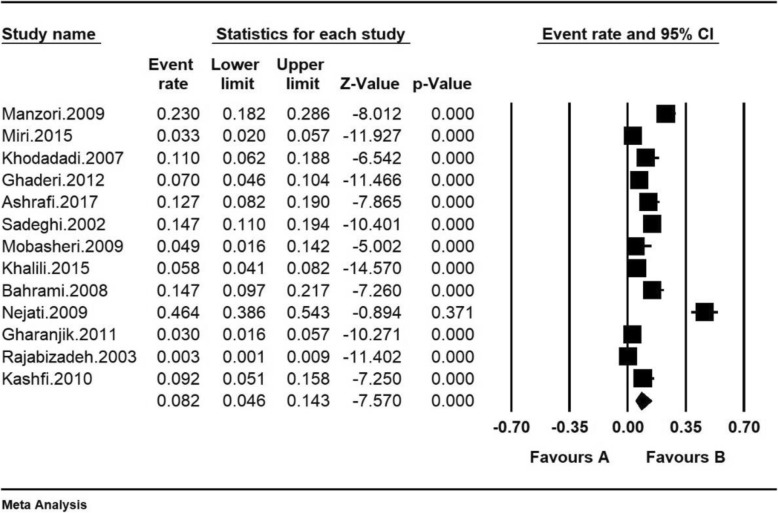
Prevalence of severe depression in Iranian older adult based on a random effects model

The total number of samples included in the study was 3948 individuals aged 50–90 years. The overall prevalence of severe depression among Iranian older adult was 8.2% (95% CI: 4.6–14.3%) according to the meta-analysis. The highest prevalence of severe depression among Iranian older adult 46.3% was seen among the older adult in Qom (95% CI: 38.6.-54.3%) in 2009 [18], and the lowest prevalence of severe depression 0.3% was also reported in the older adult in Kerman Province (95% CI: 0.1–0.9%) in 2003 [14] (Fig. 3). Fig. 3 shows the prevalence of severe depression by a random effects model, in which the black square indicates the prevalence rate and the length of the segment on which, the square is located, represents 95% CI in each study. The diamond sign indicates the prevalence rate at the national level in all studies.

#### Sub-group analysis and meta-regression

Table 2 reports the results of the sub-group analysis according to the Geographical region, year of publication, and sample size (Table 2).

**Table 2:** The results of sub-group analyses

Variables		No. studies	Prevalence% (95% CI)	I^2^ (%)	*P* value	No. participants
Geographical region	North	4	6.5 (2.8–14.5%)	89.5	0.000	925
	South	2	1.8 (0.1–3.4%)	96.9	0.000	1332
	East	–	–	–	–	–
	West	3	8.4 (5–13.9%)	61	0.000	513
	Center	4	18.7 (7.6–29.1%)	97	0.000	1178
Year of publication	2000–2006	2	2.4 (0.1–54.1%)	–	–	1491
	2007–2010	6	15.9 (8.2–28.6%)	99	0.000	816
	> 2011	5	5.7 (3.5–9.1%)	98.2	0.000	1641
Sample size	< 200	6	14 (6.3–28.4%)	99.5	0.000	718
	200–400	5	7.9 (3.6–16.6%)	96.2	0.000	1518
	> 400	2	1.5 (0.1–20.6%)		0.000	1712

**Fig. 4:**
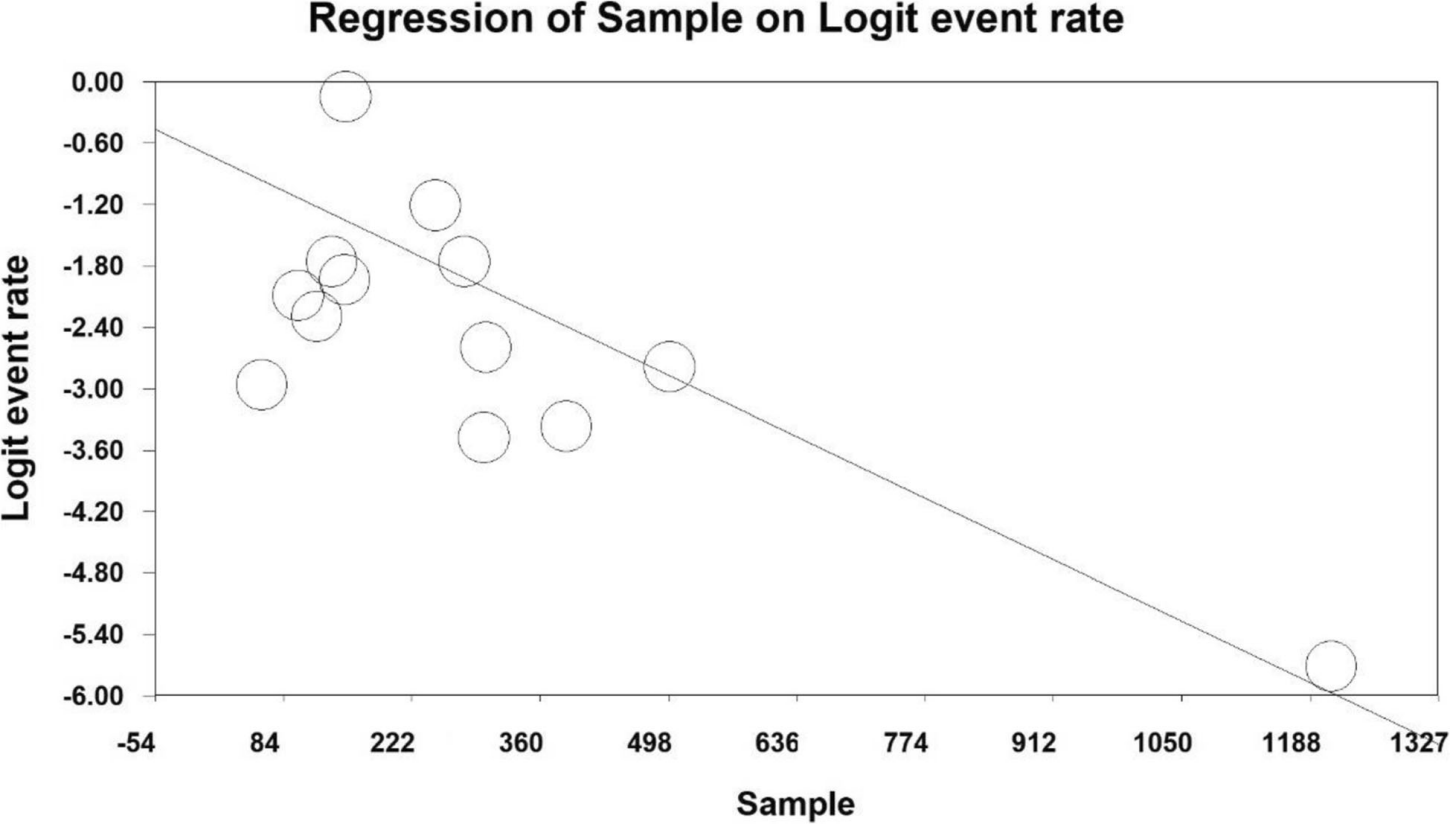
A meta-regression chart of the prevalence of severe depression in Iranian older adult by sample size

**Fig. 5:**
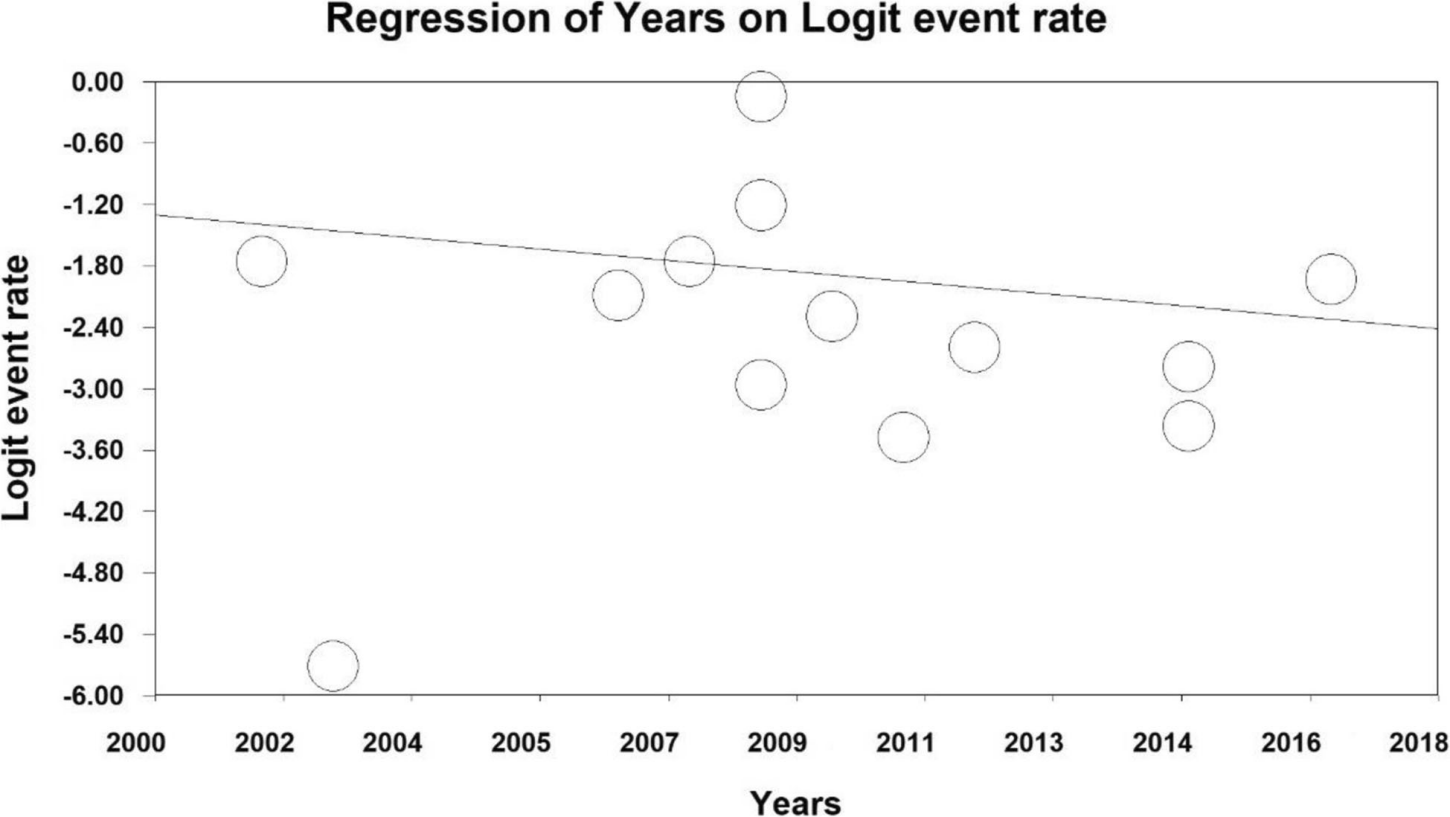
A meta-regression chart of the prevalence of severe depression in Iranian older adult by year of study

**Fig. 6:**
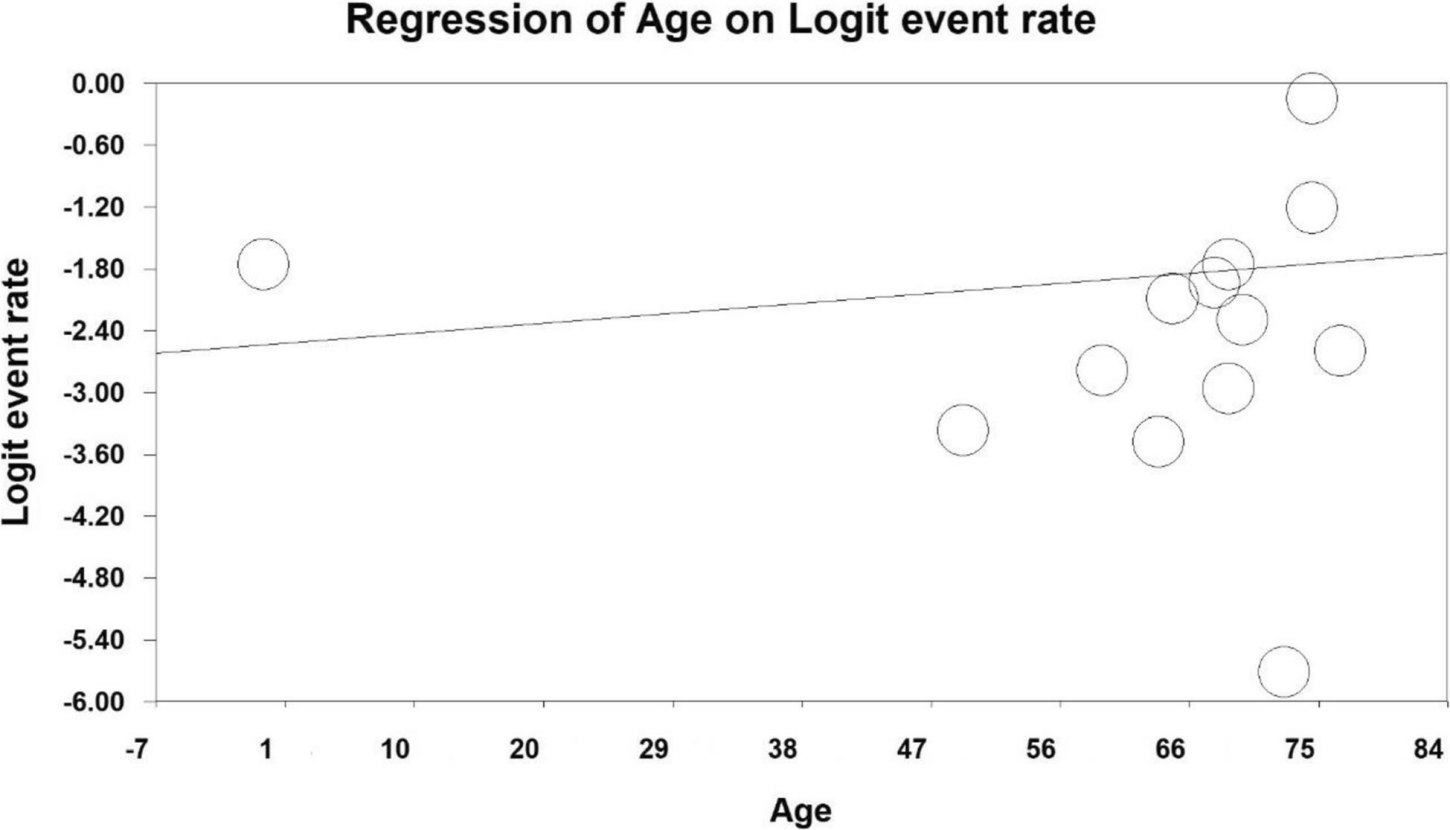
A meta-regression chart of the prevalence of severe depression in Iranian older adult by age of study participants

In order to investigate the effects of potential contributing factors on the heterogeneity of studies on prevalence of severe depression in Iranian older adult, the meta-regression test was used to study the three factors of sample size, year of study and age of study participants (Figs. 4, 5 and 6). With increasing sample size and year of study, the prevalence of severe depression in Iranian older adult decreases, which is statistically significant (Figs. 4 and 5) (*P* < 0.05) and With increasing age of study participants, the prevalence of severe depression in Iranian older adult increases, which is statistically significant (Fig. 6) (*P* < 0.05).

## Discussion

The results of the present study and investigation of 3948 people aged 50–90 years, the overall prevalence of severe depression in Iranian older adult was reported to be 8.2% based on a meta-analysis. Results of a meta-analysis study showed the overall prevalence of depression in the older adult was 43% in Iran between 2001 and 2015 [26]. A study in Sweden showed the prevalence of depression was 4.2% and the moderate-severe type was 1.6% [27]. A study in China also showed that the overall prevalence of depression in the older adult was 36.9%, and reported that the prevalence of symptoms was higher in women 50.4% as compared to men 33.3%. In general, the prevalence of moderate to severe depression was 3.9% in their study [28]. The prevalence of mild-moderate depression and mild-severe depression was reported to be 27, and 12% in a study in Greek, respectively [29]. Another study in China reported that 26.5 and 4.4% of older adult people with depression had mild and severe depression, respectively [30].

Depression is often not diagnosed in the older adult and has important impacts on quality of life, clinical outcomes, functional status, medical services, mortality, and disability [31]. Depression in the older adult also leads to increased drug use, increase costs for drugs and over-the-counter drugs, higher risk of alcohol abuse, increased length of stay, and cost of care [32]. Depression occurs in the older adult, similar to younger people due to socio-psychological and biological factors [33]. Depression is a relapsing persistent disease, and risk factors for depression in older adult people include social isolation, marital status, divorce or separation, low socioeconomic status, debilitating comorbidities, insomnia, and functional and cognitive disorders [34].

Based on the results of various studies it was reported that depression in older adult had a significant relationship with married statues, satisfaction with place of living, use of medicine, living with spouse and children, emotional support of family, members, emotional support of friends, emotional support of others, satisfaction with relations and satisfaction with overall support [15, 19, 23].

These studies also reported that Prevalence of mental disorders among older age, female, low education, marital status, activity, and income, unemployed and disabled older adult was significantly [19, 20, 25].

Increased rates of depression in the older adult, especially the severe type reported in this study, are often neglected, making it difficult to diagnose and treat the disease in a timely manner. This is an unfortunate reality because depression is a disorder for which effective treatments are available today. The results of interventional studies Legrand et al. [35] and Koshyar et al. [36] were indicated in the reducing depression: regular physical activity and appropriate diet were Reduced the level of depression, These studies reported that both outdoors and indoors 15-min self-paced walks and Participating in enjoyable and pleasant exercises were associated with significant and positive affective, There is scientific agreement that exercise temporarily makes people feel more positively activated so that more frequent exercise may lead to a greater cumulative effect on positive affect and perhaps more opportunities to reduce negative thoughts and ruminative processes [35, 36].

It is also worth noting that the reduced mental ability and the feeling of sadness is not part of the normal aging process and this mood state should be considered important [34, 37]; therefore, considering the foregoing, screening and secondary prevention measures by nurses and healthcare providers are of great importance. Moreover, increasing families’ awareness of depression in the older adult will also pave the way for early diagnosis and more appropriate treatment.

## Limitations

The most important limitations of the current study include the lack of access to full text and the poor quality of some of the studies studied.

## Conclusion

Considering the high prevalence of severe depression in the Iranian older adult, it is necessary for health policy makers to take effective control measures including periodic care for the older adult.

## Data Availability

Datasets are available through the corresponding author upon reasonable request.

## Abbreviations

PRISMA: Preferred reporting items for systematic reviews and meta-analysis
SID: Scientific information database
STROBE: Strengthening the reporting of observational studies in epidemiology for cross- sectional study
WHO: World health organization
